# Contrasting responses of steppe *Stipa* ssp. to warming and precipitation variability

**DOI:** 10.1002/ece3.5452

**Published:** 2019-07-15

**Authors:** Xiaomin Lv, Qijin He, Guangsheng Zhou

**Affiliations:** ^1^ State Key Laboratory of Severe Weather Chinese Academy of Meteorological Sciences Beijing China; ^2^ College of Resources and Environmental Sciences China Agricultural University Beijing China; ^3^ Collaborative Innovation Center on Forecast Meteorological Disaster Warning and Assessment Nanjing University of Information Science & Technology Nanjing China

**Keywords:** climatic warming, critical water threshold, Inner Mongolia steppe, precipitation variability, sensitivity

## Abstract

Climate change, characterized by warming and precipitation variability, restricted the growth of plants in arid and semiarid areas, and various functional traits are impacted differently. Comparing responses of functional traits to warming and precipitation variability and determining critical water threshold of dominate steppe grasses from Inner Mongolia facilitates the identification and monitoring of water stress effects. A combination of warming (ambient temperature, +1.5°C and +2.0°C) and varying precipitation (−30%, −15%, ambient, +15%, and +30%) manipulation experiments were performed on four *Stipa* species (*S. baicalensis*, *S. bungeana*, *S. grandis*, and *S. breviflora*) from Inner Mongolia steppe. The results showed that the functional traits of the four grasses differed in their responses to precipitation, but they shared common sensitive traits (root/shoot ratio, R/S, and specific leaf area; SLA) under ambient temperature condition. Warming increased the response of the four grasses to changing precipitation, and these differences in functional traits resulted in changes to their total biomass, with leaf area, SLA, and R/S making the largest contributions. Critical water thresholds of the four grasses were identified, and warming led to their higher optimum precipitation requirements. The four steppe grasses were able to adapt better to mild drought (summer precipitation decreased by 12%–28%) when warming 1.5°C rather than 2.0°C. These results indicated that if the Paris Agreement to limit global warming to 1.5°C will be accomplished, this will increase the probability for sustained viability of the *Stipa* steppes in the next 50–100 years.

## 1 INTRODUCTION

Global climate projection models suggest that spatial and temporal patterns of temperature and precipitation will become increasingly heterogeneous, with arid areas in the lower latitudes seeing more frequent and severe droughts. Warm and dry climates are increasingly prevalent across much of China (Tan et al., 2017) and have seriously affected the terrestrial ecosystem and food security (Lesk, Rowhani, & Ramankutty, 2016; Reichstein et al., 2013; Sun et al., 2017). Studies have revealed a greater frequency of droughts in the semiarid areas of northern China (Wu, Dijkstra, Koch, Peñuelas, & Hungate, 2011), which will become even more common in the future as a result of climate change (Battisti & Naylor, 2009; Ma, Zhou, Angélil, & Shiogama, 2017). Steppe has an important strategic position for ecological security in China, covering an area of about 4 × 108 ha, or about 41.67% of the territory of China. Steppes in Inner Mongolia account for nearly 67% of the total area of temperate steppe in China (Kang, Han, Zhang, & Sun, 2007) and are highly affected by climate change (Sui, Zhou, & Zhuang, 2013). Decreases in water availability have led to the migration of steppe vegetation and a reduction in steppe area, with additional changes to the structure and productivity of steppe plant communities (Wertin, Reed, & Belnap, 2015; Wilcox, Fischer, Muscha, Petersen, & Knapp, 2015). These impacts have been exacerbated by increasing ambient temperatures (Castagneri, Regev, Boaretto, & Carrer, 2017; Li et al., 2016).

Plant functional traits are the internal or external adaptive characters formed by the interaction of plants and environment (Salgado‐Negret, Canessa, Valladares, Armesto, & Perez, 2015). Previous studies have revealed that mild decreases in precipitation do not significantly affect plant growth; however, extreme drought significantly reduces plant height, leaf area, and photosynthetic capacity, negatively impacting the production capacity of the plants (Bret‐Harte et al., 2001; Cowling et al., 2015; Munson et al., 2013; Phillips et al., 2009; Shi, Zhou, Jiang, Wang, & Xu, 2016; Xu et al., 2014). For the scientific management of plant resources in our changing climate, a detailed comparison of the responses of functional traits to precipitation variability in different steppe grasses is needed. This will help to guide the application of drought control and resistance methods in a timely manner (Nepstad et al., 2004). Biomass reflects the growth and resource utilization of plants and is an important research focus of steppe ecology (Manea, Sloane, & Leishman, 2016). Plants respond to reduced precipitation mainly by inhibiting gas exchange while concurrently adjusting their physiological characteristics, thereby altering plant functions such as net primary production or biomass (Brodribb, Bowman, Nichols, Delzon, & Burlett, 2010; Li, Zhao, & Liu, 2015; Shafran‐Nathan, Svoray, & Perevolotsky, 2013). In addition, differences in the functional traits to precipitation variability mean that the final total biomass of a plant is not necessarily altered, which reflects the adaptive strategies of the plant species to changing environments (Brunner, Herzog, Dawes, Arend, & Sperisen, 2015; Chapin, Bloom, Field, & Waring, 1987). However, quantitatively assessing the contribution of various functional traits to biomass has drawn little attention to date.

In response to precipitation variability, plants are either adaptive or stressed; however, even resilient plants have a limited capability to adapt to reduced precipitation, and the growth and survival of plants are inhibited by prolonged or extreme changes in precipitation that exceed their adaptation and recovery abilities (Barkaoui, Navas, Roumet, Cruz, & Volaire, 2017; Sankaran et al., 2005). The determination of these limits confirms the drought threshold of plant and is essential for the application of drought control and resistance methods. Especially, the response range of the dominate plant species to precipitation variability is particularly representative of the adaptability of the natural ecosystem (Cavin, Mountford, Peterken, & Jump, 2013; Waddington et al., 2015). Identifying critical precipitation thresholds of biomass in dominant steppe species to precipitation variability is therefore important for the evaluation of the effects of climate change on these ecosystems (IPCC, 2014; Palumbi, Barshis, Traylor‐Knowles, & Bay, 2014). A recent study revealed that a linear model was not applied to describe temporal precipitation—aboveground net primary production (ANPP) relationships, but a nonlinear form of the temporal precipitation—ANPP relationship would better predict responses of ANPP to changing precipitation regimes because the precipitation variability and extreme precipitation were forecast to increase with climate change (Knapp, Ciais, & Smith, 2017). *Leymus chinensis* and *S. grandis* reached their peak biomass when the relative soil moisture was 66% and 54.7%, respectively, via a simulated experiment (Xu & Zhou, 2011); thus, the biomass decreased with decreasing precipitation and there existed the response threshold to drought. But warming strengthened the negative effects of drought to plant biomass (Hoover, Knapp, & Smith, 2016; Yang, Wang, Yang, & Guo, 2013). These studies showed that biomass responded differently to precipitation in different species and more focused on the responding extent of plants to precipitation variability. Few studies have reported the critical precipitation threshold of biomass at different warming levels.

*Stipa* L. dominates the zonal steppe communities of Inner Mongolia, presenting a regular zonal distribution from east to west in the steppes (Shi et al., 2016; Song, Wang, & Lv, 2016). Among them, *S. baicalensis* dominates in semiarid and semihumid meadow steppes, *S. bungeana* grows in the semiarid warm temperate typical steppes, *S. grandis* grows in the semiarid typical steppe, and *S. breviflora* dominates in the arid and semiarid desert steppes (Qi et al., 2010). Here, these four representative *Stipa* species as subjects in climate simulation experiments under ambient and warming conditions with precipitation variability. The main objectives of this study were to identify difference in responses of functional traits and drought threshold to precipitation variability at different warming levels. Three hypotheses were tested as follows: (a) Among the functional traits, the growth strategy traits (root/shoot ratio, R/S) may be more sensitive to precipitation than the other traits and warming may improve the responses of those traits; (b) warming may lead to higher optimum water requirements for total biomass and critical water threshold may differ in the four *Stipa* species; (c) *Stipa* species may be able to adapt better to drought when temperatures were increased by 1.5°C rather than 2.0°C. These results will help to identify and monitor the impacts of drought, and provide evidence for dealing with drought in Inner Mongolia steppe.

## 2 MATERIAL AND METHODS

### 2.1 Experiment design and environmental conditions

**Figure 1:**
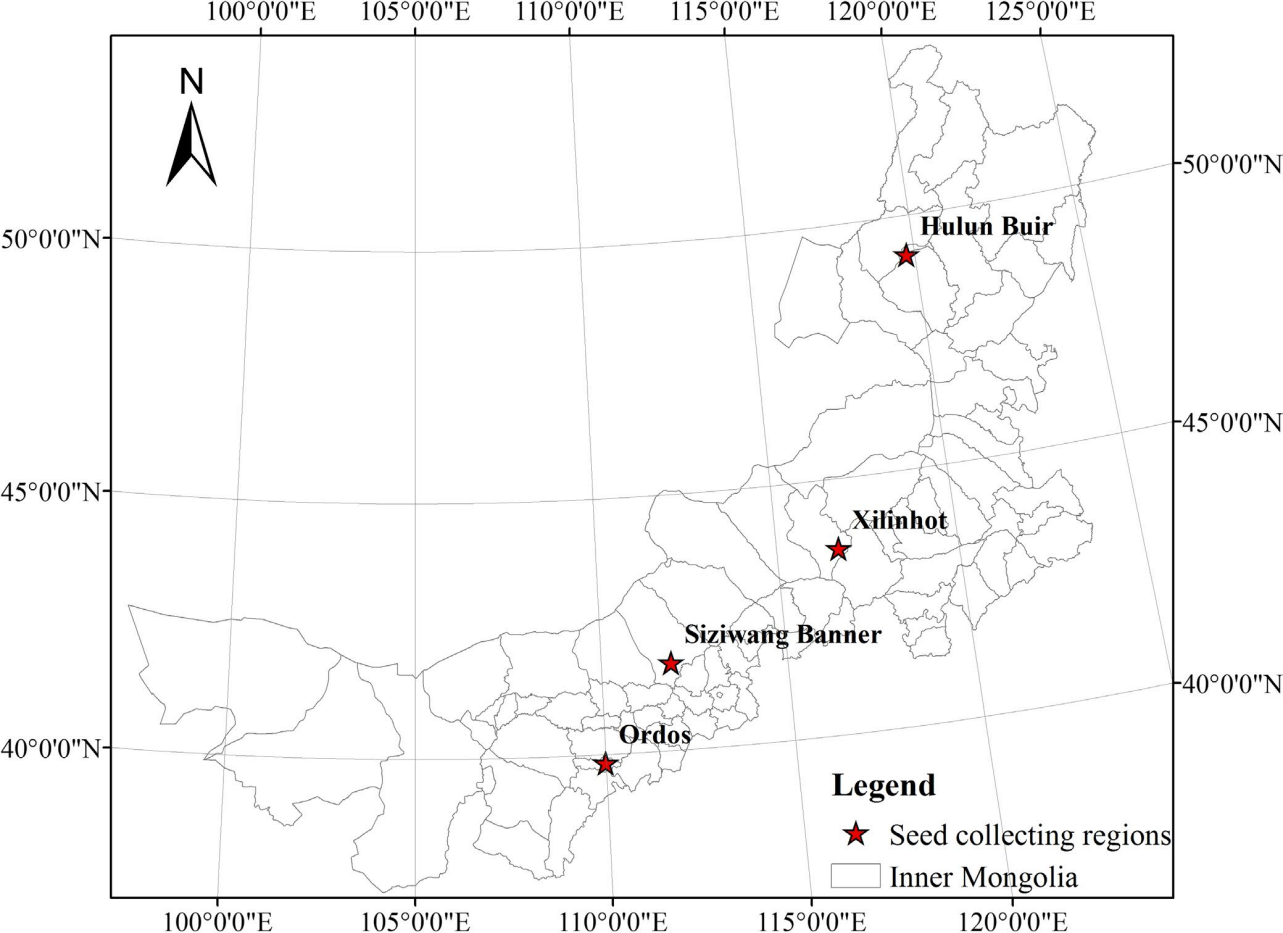
The site of seed collecting in the four *Stipa* species

The manipulation experiment was conducted in the Institute of Botany, Chinese Academy of Science from the November 2011 to September 2012. Seeds of *S. baicalens*, *S. grandis*, *S. bungeana*, and *S. breviflora* were collected 1 year ahead of experiment from natural steppe in Hulun Buir (49°13′N, 119°45′E), Xilinhot (43°57′N, 116°07′E), Siziwang Banner (41°43′N, 111°52′E), and Ordos (39°50′N, 109°59′E), respectively (Figure 1). All the seeds were sterilized with 5% potassium permanganate and subsequently washed with water before sowing. The polyethylene pots (10.9 cm in diameter, 9.5 cm in height, 0.71 L in total volume) were used as the experimental containers, which were wrapped with plastic film to prevent water leakage. Each pot was filled with approximately 0.61 kg of dry chestnut soil (organic carbon content 12.3 g/kg, total nitrogen content 1.45 g/kg, and soil field capacity 24.8%–26.8%), and 10 seeds were planted per pot. All pots were first placed in glasshouse (day/night temperature of 26–28/18–20°C, maximum photosynthetic photon flux density of 1,000 mol m^–2^ s^–1^) and well‐watered to complete the growth of the seedlings (Lv, Zhou, Wang, & Song, 2016; Song et al., 2016). Until the third leaf emergence (about 3 weeks after sowing), the seedlings were thinned to four plants per pot. Six replicates were used for the five precipitation and the three temperature treatments. Then, the 90 pots (four plants per pot) per *Stipa* plant were randomly transferred into three climate control chambers (RXZ‐500D, The southeast instruments Inc.). Different temperature and water treatments were set based on the average monthly temperature (T0) and water (W0) during the four species' own growth stage in the past 30 years (1978–2007), which were shown in Tables 1 and 2. Three temperature treatments were set: (ambient temperature (T0), +1.5°C (T1), +2.0°C (T2). Five water treatments were set: average monthly water over 30 years (W0), the average decreased by 30% and 15% (W1 and W2), and the average increased by 15% and 30% (W3 and W4). During the experiment, precipitation was added to the pots by a sprayer every 3 days, as described in our previous similar experiments (Shi et al., 2016; Xu et al., 2014).

**Table 1:** The ambient temperatures during the four *Stipa* species' own growth stage in the past 30 years (1978–2007)

Species	June		July		August	
	*T*_d_/*T*_n_ (°C)	*T*_m_ (°C)	*T*_d_/*T*_n_ (°C)	*T*_m_ (°C)	*T*_d_/*T*_n_ (°C)	*T*_m_ (°C)
*S. bungeana*	22.4/16.4	19.4	24.3/18.3	21.3	22.3/16.3	19.3
*S. breviflora*	22.4/16.4	19.4	24.3/18.3	21.3	22.3/16.3	19.3
*S. grandis*	22.1/16.1	19.2	24.4/18.4	21.4	22.6/16.6	19.6
*S. baicalens*	21.0/15.0	18.0	23.2/17.3	20.3	21.2/15.2	18.2

Note

*T*_d_/*T*_n_, average daytime/nighttime temperature, and *T*_m_, monthly average temperature.

**Table 2:** The ambient precipitation during the four *Stipa* species' own growth stage in the past 30 years (1978–2007) and the water regimes

Species	Months	Precipitation (mm)				
		−30%	−15%	Ambient	+15%	+30%
*S. bungeana*	June	36	43	51	59	66
	July	68	82	97	112	126
	August	69	84	99	114	129
	Total	173	210	247	284	321
*S. breviflora*	June	36	43	51	59	66
	July	68	82	97	112	126
	August	69	84	99	114	129
	Total	173	210	247	284	321
*S. grandis*	June	32	38	45	52	59
	July	55	66	78	90	101
	August	46	55	65	75	85
	Total	132	160	188	216	244
*S. baicalens*	June	39	47	55	63	72
	July	66	80	94	108	122
	August	63	77	90	104	117
	Total	167	203	239	275	311

### 2.2 Soil water content measurement

The soil relative water content (SRWC, %), the ratio between the current soil moisture and the field capacity, was measured using the gravimetric method from 0 to 10 cm soil layer. The measurement was determined 90 days after the plants were subjected to a relative long‐term soil water treatment. Six replicates were used for SWC determination. The SRWC is calculated as follows:

(1) $$\text{SRWC}=\frac{\text{SWC}}{\text{FC}}\times 100=\frac{\left(W_{c}-W_{d}\right)/\left(W_{d}-W_{p}\right)}{\text{FC}}\times 100,$$

where *W*_c_ is the weight for empty soil pot and wet soil (g), *W*_p_ is the empty soil pot weight (g), *W*_d_ is the weight for empty soil pot and dry soil (g), and FC is the soil field capacity (%).

### 2.3 Plant measurements

At the end of the experiment, all pots of each treatment were harvested to measure response characteristics (morphological characteristics and biomass). Leaf number and plant height were measured before harvest. Leaf area per plant was measured with a WinFOLIA system to measure the area of the blade part per plant (WinRHIZO, Regent Instruments). Plants were separated into three parts to harvest: leaf, stem, and root, and dried at 80°C to a constant weight, then weighted to get aboveground (leaf and stem) and belowground (root) biomass separately. Growth indices per plant were calculated as follows: specific leaf area (SLA; cm^2^/kg) = leaf area/leaf biomass; leaf area ratio (LAR; cm^2^/kg) = leaf area/plant total biomass; and root–shoot ratio (R/S) = belowground biomass/aboveground biomass.

### 2.4 Statistical analysis

A method for assessing sensitivity was applied to study the differed responses of functional traits to precipitation variability in the four *Stipa* species. The response coefficient, which also could be called plasticity index, was defined as the absolute value of the relative change in a functional trait per unit of precipitation change compared with ambient precipitation level. A higher response coefficient (plasticity index) indicates a more response sensitivity to precipitation variability of this functional trait (Luers, 2005).

The tipping point of the four *Stipa* species responding to precipitation variability is defined as the very point when the total biomass starts to deviate significantly from those with sufficient water supply, resulting from a decline in precipitation amount below a critical level (Czajkowski, Ahrends, & Bolte, 2009; Thompson, Gallardo, Valdez, & Fernández, 2007). This method named as one‐side lower tolerance limits for normal population was newly adopted to identify the tipping point of total biomass in the four species. The tolerance interval is an estimated interval within which at least a certain proportion *β* of the population falls at a given level of confidence γ; tolerance limit refers to either of the two endpoints of a tolerance interval (Krishnamoorthy & Mathew, 2009; Shi et al., 2016; Xu et al., 2009; Young, 2014). The rigorous statistical definitions could be found in Young (2014). Computational formulas of tolerance intervals and limits for different distribution populations are provided by ISO 16269‐6(2005) (Xu et al., 2009). Given the sampling methods of the experiment, formulas of one‐side lower tolerance limits for normal population with unknown variance and unknown mean were calculated as follows:

(2) $$x_{L}=\bar{x}-k\left(n;1-\beta ;1-\gamma \right)\times s$$

(3) $$\bar{x}=\sum x/n$$

(4) $$s=\sqrt{\frac{n\sum x^{2}-{\left(\sum x\right)}^{2}}{n\left(n-1\right)}}$$

where *x_L_* is one‐side lower tolerance limits for total biomass; $\bar{x}$ is the sample mean; *s* is the standard deviation of the sample variance; *n* is the sample size; *x* is the sample value; 1‐*β* is the least proportion of sample population within the tolerance interval, defined as 95%; 1‐*γ* is the confidence level, defined as 95%; and *k* is the tolerance coefficient, which could refer to ISO 16269‐6 (2005) (Xu et al., 2009) or be calculated directly (Krishnamoorthy & Mathew, 2009).

The relationship between total biomass and precipitation under different temperatures was analyzed using a regression analysis. The tipping points of total biomass in the four species were further quantified by their values of critical water thresholds, which were calculated in terms of the tipping points and the quadratic polynomial regression models between total biomass and precipitation under ambient and warming temperature treatments for the four species (Equation 5).

(5) $$X=a\times {\text{Pre}}^{2}+b\times \text{Pre}+c$$

where *X* is the observed values of the total biomass under different temperature treatment for the four species; Pre is the precipitation amount (mm); and *a*, *b*, and *c* are the fitting coefficients of the regression model.

All collected data were processed using the statistical software SPSS 17.0 (SPSS) and Origin 9.0 (Origin Lab). Two‐way analyses of variance (ANOVAs) were used to examine the effects of warming and precipitation variability. The differences between the means among the precipitation treatments were compared using Duncan's multiple range tests. The effects of different response characteristics on the plant biomass were analyzed using principal component and pathway analyses. All statistical significances were denoted at *p* < .05 unless otherwise noted.

## 3 RESULTS

### 3.1 Changes in relative soil water content under warming and varying water

**Figure 2:**
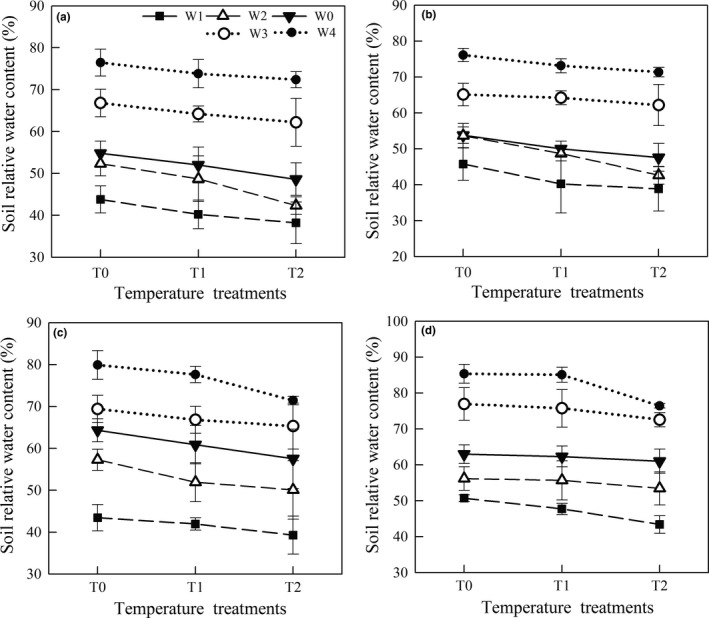
Changes in soil relative water content of the four *Stipa* plants under different temperature and water treatments. Vertical bars represent ±*SE* of the mean (*n* = 6). (a) *S. breviflora*, (b) *S. bungeana*, (c) *S. grandis*, (d) *S. baicalensis*

Under the same temperature treatment, the changes in SRWC were associated with water regimes in the four *Stipa* plants (Figure 2). Under every temperature treatment, increased water treatments (W3 and W4) were in favor of increasing SRWC, but decreased water reduced SRWC. Likewise, SRWC decreased with increased temperature under the same water treatment in the four species (Figure 2).

### 3.2 Sensitivity of functional traits to precipitation variability under warming

**Figure 3:**
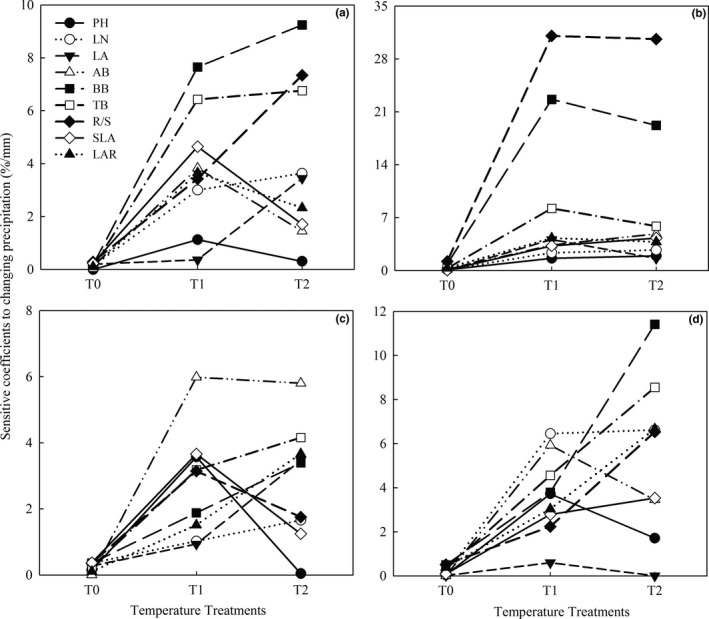
Response coefficients of the four *Stipa* species to precipitation variability under different temperature treatments. (a) *S. breviflora*, (b) *S. bungeana*, (c) *S. grandis*, (d) *S. baicalensis*. T0, T1, and T2 denote increasing temperature by 0, 1.5, and 2.0°C, respectively, relative to the average temperature of the growing season over past 30 years in the four *Spita* plants

A variance analysis revealed that changes in precipitation significantly affected the plant height (PH), leaf area (LA), aboveground biomass (AB), underground biomass (BB), total biomass (TB), and leaf area ratio (LAR) in all four *Stipa* species (*p* < .05, Table 3). Leaf number (LN) of *S. baicalensis*, R/S of *S. breviflora*, and specific leaf area (SLA) of *S. grandis* and *S. baicalensis* were not significantly affected by precipitation. The responses of various functional traits to precipitation variability also differed significantly under different warming condition. Under ambient temperature treatment (T0), the responses of functional traits in all four species to precipitation changes were relatively low; R/S was the most sensitive trait in *S. bungeana*, *S. grandis*, and *S. baicalensis* (Figure 2b–d), while SLA was the most sensitive trait in *S. breviflora* (Figure 3a). Compared with ambient temperature, warming 1.5°C (T1) and 2.0°C (T2) enhanced the responses of the *Stipa* species to precipitation variability. Under T1 and T2 conditions, the most significantly affected (and therefore most sensitive traits to precipitation variability) were BB, R/S, and AB in *S. breviflora*, *S. bungeana*, and *S. grandis*, respectively. The most sensitive traits in *S. baicalensis* were LA under T1 and BB under T2 condition (Figure 3).

**Table 3:** ANOVA analysis (*F* value) of the effects of precipitation on different functional traits

Species	PH	LN	LA	AB	BB	TB	R/S	SLA	LAR
*S. breviflora*	18.12**	9.52**	3.75**	20.29**	10.65**	18.91**	1.84	5.45**	4.39**
*S. bungeana*	14.74**	17.51**	30.89**	46.50**	11.51**	32.02**	5.36**	9.25**	3.63*
*S. grandis*	4.54**	9.21**	11.20**	21.00**	36.43**	51.35**	4.29**	0.74	3.56*
*S. baicalens*	2.45*	0.72	3.57**	10.64**	11.22**	16.25**	4.46**	1.44	6.52**

* and ** represent statistically significant at *p* < 0.05 and 0.01.

According to their response coefficients of traits in response to precipitation variability under different temperature treatments, the order of response traits for the four species under T0 was *S. bungeana* > *S. baicalensis* > *S. grandis* > *S. breviflora*. Under T1 and T2 treatments, *S. bungeana* still had the greatest response to precipitation among the four species, but *S. grandis* had the lowest response, and those of *S. baicalensis* and *S. breviflora* were similar (Table 4).

**Table 4:** Maximum response coefficients of functional traits to varying precipitation under different temperature treatments in *Stipa* species

Maximum response coefficients (%/mm)	Species			
	*S. baicalens*	*S. grandis*	*S. breviflora*	*S. bungeana*
T0	0.51	0.38	0.29	1.21
T1	6.46	5.98	7.65	31.06
T2	11.42	5.80	9.25	30.63

Note

T0, T1, and T2 denote increasing temperature by 0, 1.5, and 2.0°C, respectively, relative to the average temperature of the growing season over past 30 years in the four *Spita* plants.

### 3.3 Relationship between various functional traits and total biomass

**Figure 4:**
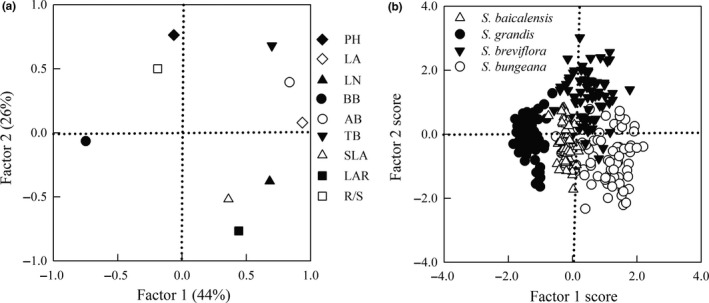
Principal component analysis on the four *Stipa* species. (a) The first two principal components (PCs) and the pattern of characteristic loadings, (b) the projections of two PCs sorted by the four *Stipa* species

Based on a principal component analysis of functional traits in the four species, the variance explained by principal components 1 (production capacity) and 2 (growth strategy) was 44% and 26%, respectively, meaning that these two factors accounted for 70% of the variation arising from the nine variables investigated (Figure 4a). The production capacity was closely related to BB, AB, LA, and LN. The growth strategy was closely related to the PH, SLA, LAR, and R/S. TB was closely related to both of these first and second principal components. The four *Stipa* species could be distinguished by their score for these two principal components (Figure 4b); for the first factor, production capacity, *S. bungeana* had the highest score, while *S. grandis* had the lowest score. *S. breviflora* scored highest in the second factor, growth strategy, while *S. bungeana* scored lowest.

Based on a stepwise regression analysis of the functional traits, LA, SLA, and R/S were determined as the main impact factors to the total biomass of *S. bungeana*, *S. grandis,* and *S. breviflora*, while LA, R/S, and LAR impacted significantly the biomass of *S. baicalensis* (Table 5). Through a pathway analysis, R/S was most closely correlated with the total biomass, with correlation coefficients of .49, .61, and .85, respectively, in *S. breviflora*, *S. bungeana,* and *S. baicalensis*. LA was most closely correlated with total biomass in *S. grandis* (correlation coefficient: .60). Other main factors had various direct and indirect effects on the total biomass of the *Stipa* species. The functional traits with the highest direct contributions to total biomass of *S. bungeana*, *S. breviflora*, *S. grandis*, and *S. baicalensis* were LA (0.75), LA (0.81), SLA (−0.79), and LAR (−0.80), respectively, while the traits with highest indirect contributions were SLA, LA, SLA, and R/S, respectively (Table 5).

**Table 5:** Pathway analysis for main impact factors to total biomass in the four *Stipa* species (— indicates none)

Species	Independent variable	Correlation coefficient	Direct contribution rate	Indirect contribution rate				
				LA	SLA	R/S	LAR	Total
*S. breviflora*	LA	.324	0.748	—	−0.678	0.259	—	−0.419
	SLA	−.231	−1.044	0.485	—	0.330	—	0.815
	R/S	.486	0.699	0.277	−0.493	—	—	−0.216
*S. bungeana*	LA	.246	0.808	—	−0.506	−0.055	—	−0.561
	SLA	−.225	−0.748	0.546	—	−0.023	—	0.523
	R/S	.607	0.650	−0.069	0.026	—	—	−0.043
*S. grandis*	LA	.600	0.764	—	−0.258	0.094	—	−0.164
	SLA	−.193	−0.788	0.250	—	0.345	—	0.595
	R/S	.302	0.623	0.115	−0.437	—	—	−0.321
*S. baicalens*	LA	.241	0.441	—	—	0.019	−0.218	−0.200
	R/S	.848	0.204	0.040	—	—	0.604	0.644
	LAR	−.831	−0.796	0.121	—	−0.156	—	−0.035

### 3.4 Critical water threshold of the four *Stipa* species

#### 3.4.1 Effects of different precipitation treatments on total biomass

**Figure 5:**
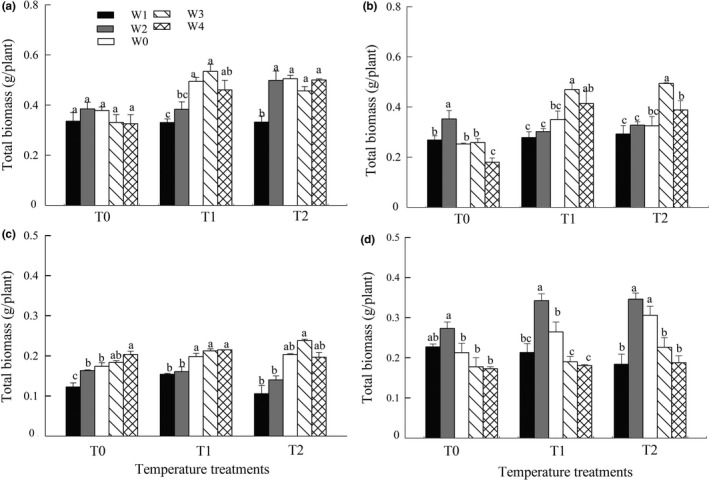
Effects of precipitation variability on total biomass of the four grasses under different temperature treatments. Data: mean ± *SE*; (a) *S. breviflora*, (b) *S. bungeana*, (c) *S. grandis*, (d) *S. baicalensis*. Different letters indicate significant differences among the precipitation treatments within the same temperature treatment

Duncan multiple comparisons showed that, under T0 treatment, the different precipitation treatments did not influence the total biomass of *S. breviflora,* while the total biomass of *S. bungeana*, *S. grandis*, and *S. baicalensis* was significantly lower under the treatment of W1 and W2 (Figure 5). Under T1 and T2 treatments, the total biomass of *S. breviflora* treated with −30% precipitation (W1) was significantly lower than in the other precipitation treatments; in *S. bungeana* and *S. grandis*, the total biomass was significantly lower in decreased precipitation treatments (W1 and W2) than in increased precipitation treatments; the total biomass of *S. baicalensis* treated with −30% precipitation (W1) was significantly lower than under the −15% and control precipitation (W2 and W0) conditions (Figure 5).

#### 3.4.2 Relationship of total biomass and precipitation in the four *Stipa* species

**Figure 6:**
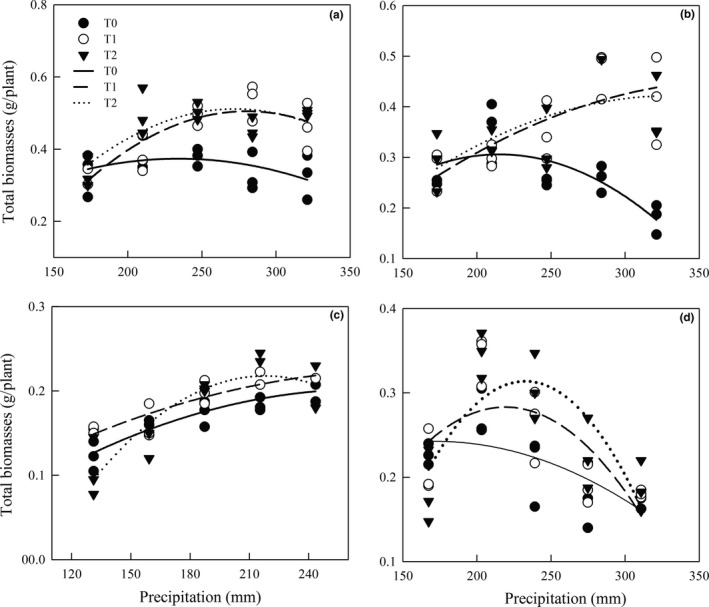
Relationship of total biomass with precipitation under different temperature treatments in the four *Stipa* species. (a) *S. breviflora*, (b) *S. bungeana*, (c) *S. grandis*, (d) *S. baicalensis*

Under T0 treatment, there was a quadratic curve relationship between the total biomass of *S. bungeana* and *S. grandis* and the precipitation rate (*p* < .05), but no significant relationship was observed for *S. breviflora* and *S. baicalensis* (*p* > .05). Under warming conditions (T1 and T2), the total biomass of the four *Stipa* species all showed a significant quadratic curve relationship with changing precipitation (*p* < .05, Table 6 and Figure 6).

**Table 6:** Relationships between total biomass and precipitation under different temperature treatments in the four *Stipa* species

Species	Temperature treatments	Regression equation	*R^2^*	*p* Values
*S. breviflora*	T0	*y *= −7.67 × 10^−6^ *x* ^2^ + 0.0036*x *− 0.0517	.18	.305
	T1	*y *= −1.68 × 10^−5^ *x^2^* + 0.0094*x *− 0.8108	.70	.001
	T2	*y *= −1.56 × 10^−5^ *x^2^* + 0.0085*x *− 0.6464	.57	.006
*S. bungeana*	T0	*y* = −1.15 × 10^−5^ *x^2^* + 0.0049*x *− 0.2253	.56	.007
	T1	*y *= −4.46 × 10^−5^ *x^2^* + 0.0034*x *− 0.1901	.57	.007
	T2	*y *= −5.0 × 10^−6^ *x^2^* + 0.0038*x *− 0.2052	.41	.043
*S. grandis*	T0	*y *= −3.94 × 10^−6^ *x^2^* + 0.0021*x *− 0.0840	.83	.000
	T1	*y *= −2.87 × 10^−6^ *x^2^* + 0.0017*x *− 0.0234	.81	.000
	T2	*y *= −1.63 × 10^−5^ *x^2^* + 0.0071*x *− 0.5555	.79	.000
*S. baicalensis*	T0	*y *= −4.24 × 10^−6^ *x^2^* + 0.0015*x* + 0.1174	.48	.265
	T1	*y *= −1.52 × 10^−5^ *x^2^* + 0.0067*x *− 0.4466	.48	.002
	T2	*y *= −2.45 × 10^−5^ *x^2^* + 0.0114*x *− 1.0111	.59	.005

### 3.5 Critical water threshold of the four species

Under ambient temperature (T0), the optimum water levels (June to August) when *S. breviflora*, *S. bungeana*, *S. grandis*, and *S. baicalensis* reached their peak total biomass were 235, 213, 266, and 172 mm (Table 7), respectively, with −28%, 41%, −5%, and −14% differences in precipitation relative to the ambient precipitation. The critical water thresholds of these species were 72, 163, 135, and 113 mm, respectively, resulting in differences in −70%, −13%, −45%, and −54% relative to their respective ambient precipitation (Table 7). The critical water thresholds of *S. breviflora* and *S. baicalensis* were not significant (*p* > .05; Tables 6 and 7).

**Table 7:** Critical water threshold analyses of total biomass under different temperature treatments in the four *Stipa* species

Species	Temperature treatments	Maximum biomasses (g/plant)	Optimum water (mm)	Threshold		
				Tipping point of total biomass (g/plant)	Critical water (mm)	The relative change in water threshold compared to ambient precipitation (%)
*S. breviflora*	T0	0.37	235	0.29	135	−45
	T1	0.51	280	0.37	190	−23
	T2	0.51	272	0.40	188	−24
*S. bungeana*	T0	0.30	213	0.18	113	−54
	T1	0.46	381	0.27	178	−28
	T2	0.52	380	0.49	312	26
*S. grandis*	T0	0.20	266	0.15	163	−13
	T1	0.23	297	0.18	165	−12
	T2	0.22	218	0.15	155	−17
*S. baicalensis*	T0	0.24	172	0.20	72	−70
	T1	0.29	220	0.26	174	−27
	T2	0.32	233	0.16	154	−36

Warming increased the optimum water levels and decreased the critical water thresholds in the four *Stipa* species. As warming, the critical water thresholds of the total biomass were −23% (T1) and −24% (T2) in *S. breviflora*; −28% (T1) and + 26% (T2) in *S. bungeana*; −27% (T1) and −36% (T2) in *S. baicalensis*; and −12% (T1) and −17% (T2) in *S. grandis* compared with the ambient water (Table 7).

## 4 DISCUSSION

Precipitation is one of the main climatic factors restricting the growth of plants in arid and semiarid areas, and various characteristics are impacted differently by changes in precipitation (Maréchaux et al., 2015; Wertin et al., 2015). This study showed that changes in precipitation significantly affect the response characteristics of four *Stipa* species in the China steppe (Table 3 and Figure 3), but differences existed between the different plant species. Under the ambient temperature treatment (T0), all the four grasses showed no or modest responses to changes in precipitation. Growth strategy traits (R/S and SLA, Figure 3) showed strongest sensitivity to changes in precipitation, indicating that *Stipa* species may adapt their growth under precipitation variability by altering their allocation of above/belowground growth under ambient temperatures, as previously reported (Chapin et al., 1987; Xu et al., 2014). *S. baicalensis*, *S. grandis*, and *S. breviflora* showed gradually declining responses to precipitation variability under the ambient temperature (Table 4), which reflected the east‐to‐west precipitation gradients in their respective native areas in the steppes of Inner Mongolia (Qi et al., 2010). Under the warming by 1.5 and 2.0°C, the responses of the four *Stipa* species to precipitation variability increased to varying degrees. *S. bungeana* was the most precipitation‐sensitive of the four species under ambient and warming (Table 4), which is probably related to its semiarid warm temperate steppe habitat and the resulting higher requirements for precipitation and temperature (Hu, Zhou, Li, Wu, & Wang, 2013). With a 1.5°C increase in temperature, the response of *S. breviflora* was more sensitive to precipitation variability than *S. baicalensis*, but the opposite with a 2.0°C increase (Table 4). So the sensitive characteristics of *S. breviflora* and *S. baicalensis* could indicate the increased range of temperature. When exposed to warming, leaf area and biomass, which reflect the production capacity, were the most sensitive traits in *S. breviflora*, *S. baicalensis*, and *S. grandis*, while R/S was the most sensitive trait in *S. bungeana* (Figure 3). This, to some extent, reflected the differences between the four species in their biological characteristics and sensitivity to precipitation, and supports the previous studies that showed that the tissues with the greatest response to changes in precipitation varied between species (Shafran‐Nathan et al., 2013; Wright et al., 2004). These findings also further indicated that the evaluation of plant responses to water stress based on a single trait is inaccurate. Species‐specific trait differences in sensitivity to changing precipitation are likely the primary reason why the four grasses occupy different ecological niches, and grow in different regions of the Inner Mongolia steppe (Cowlin et al., 2015; Donovan, Maherali, Caruso, Huber, & Kroon, 2011).

Differences in functional traits to precipitation variability not only have significant influence on the final biomass of plants, but also are a reflection of plant growth strategy to changing environment (Brunner et al., 2015; Chapin et al., 1987; Li et al., 2015). The results of a principal component analysis could explain the different trade‐off strategies of the *Stipa* species (Figure 4). This study found that the first principal component of these four species incorporated their production capacity and morphological characters and the second principal component mainly reflected the allocation capacity of resources. This reflected that the common adaptation strategy of the four *Stipa* plants in coping with hydrothermal changes may be to change the leaf size and biomass accumulation firstly, and this change will change the distribution of aboveground and underground resources, thus ensuring the maximum growth of individuals (Wright et al., 2004). The four *Stipa* plants were distributed on different scoring axes of the principal components, which indicated that the trade‐off adaptation strategies adopted between their functional traits were different when dealing with the combined changes in temperature and precipitation, and this was the reasons for different adaptability to hydrothermal changes and mainly distributed in different grassland types (Donovan et al., 2011). *S. breviflora* mainly favored the survival strategy of thick leaves and large root–shoot ratio to adapt to the arid and semiarid desert grassland environment, but *S. baicalensis* tended to choose the survival strategy of thin leaves and smaller root–shoot ratio. Both species reduced water dispersion by changing the thickness of the blade (Bret‐harte et al., 2001) and change the allocation of resources on the aboveground/underground to adapt to the larger temperature and precipitation ranges. *S. bungeana* tended to choose a strategy to reduce leaf area and increase underground growth, but *S. grandis* chose the survival strategy to increase leaf area and aboveground growth. Also, the survival strategies of the two species were also changing the allocation of resources on aboveground/underground to adapt to their corresponding living environment. Through the response of different functional traits to changes in temperature and precipitation, the four *Stipa* species would choose different trade‐off adaptation strategies to adapt to environmental changes, which further indicates that when assessing the impact of future climate change on grassland ecosystems, we should pay attention on the differences in sensitivity and adaptability of functional traits and their contribution to individual growth in responding to changing temperature and precipitation.

However, total biomass showed large variation across these two major principal components (Figure 4a), and therefore by the different responses of the various characteristics, as previously reported (Lanari, Silvestroni, Palliotti, Green, & Sabbatini, 2015; Wright et al., 2004). The functional traits that significantly influenced the total biomass of the four grasses were LA, R/S, SLA, and LAR (Table 5), mainly reflecting on the growth allocation strategy. The functional traits had different direct and indirect contributions to the total biomass of the *Stipa* plants, indicating their varied biological effects (Hu, Liu, Li, Lu, & Gao, 2014). This corresponded to the distribution of the four *Stipa* species in the different score axes of the principle component analyses, indicating that, under changes in precipitation, their different trade‐off adaptive strategies would be achieved through their response characteristics, which influence their total biomass. This was a reflection of their different adaptabilities to precipitation changes (Donovan et al., 2011; Xu et al., 2014).

Plant biomass is a combined product of photosynthesis and respiration, reflecting the comprehensive response of plants to changing environmental factors (Andresen et al., 2017; Myneni, Keeling, Tucker, Asrar, & Nemani, 1997), and is also the main research focus in steppe studies (Manea et al., 2016). Studies of the critical precipitation threshold of the four grasses would help to determine how steppe plants adapt to changing climates. This study found that the total biomass responses to changing precipitation were parabolic in *Stipa* species (Figure 6), indicating the existence of a critical point where the total biomass was significantly affected by precipitation variability. That was to say, *Stipa* plants were therefore adapted to an optimum precipitation threshold, and deviations from this value will inhibit their growth (Badger, Björkman, & Armond, 2006; Xu & Zhou, 2011). Under ambient temperatures, the optimum precipitation was lower than the actual precipitation of June to August recorded from 1978 to 2007 when the total biomass of the *Stipa* plants (except *S. grandis*) reached their highest values (Table 7), illustrating that a certain degree of precipitation reduction might not affect the growth of the *Stipa* species (except *S. grandis*). The drought thresholds of total biomass in *S. bungeana* and *S. grandis* were 113 and 163 mm, respectively, which were 54% and 13% lower than the ambient precipitation. This result corresponded to the lower limits of precipitation (104 and 179 mm) in the potential distribution areas of *S. bungeana* and *S. grandis*, calculated by Zhou, He, and Yin (2015) using the MaxEnt model. These indicated that, under ambient temperatures, a certain decrease in precipitation during the growing season (28%, 41%, 5%, and 14% decreases relative to the ambient precipitation for *S. baicalensis*, *S. grandis*, *S. breviflora*, and *S. bungeana*, respectively) can increase *Stipa* plant biomass, which may also reflect their adaptation to slightly arid environments (Fariaszewska et al., 2017). Under warming condition, the optimum precipitation (the amount of precipitation required when total biomass reaches its maximum) for total biomass in the four grasses was higher than the ambient precipitation. This may because warmer temperatures would lead to increased available soil moisture via higher evaporation rates from soil (De Boeck et al., 2008) and higher precipitation can relieve the effect of warming on these plants (Li et al., 2017). Under the warming by 1.5°C, the critical precipitation thresholds of total biomass were significantly increased in all four species. But the critical precipitation thresholds were decreased by 12%–28% (Table 7), indicating that a 1.5°C increase in temperature alongside a mild reduced precipitation would promote biomass accumulation rather than inhibit it. However, as warming 2.0°C, critical precipitation threshold of total biomass of *S. grandis* and *S. baicalensis* was decreased by 17% and 38%, respectively, compared with these in warming 1.5°C, while their critical precipitation thresholds were decreased by 6% and 11%. Also, the critical precipitation threshold of *S. bungeana* was increased by 75% relative to T1 condition and did not change significantly in *S. breviflora* (Table 7). Thus, *Stipa* plants can better adapt to a certain degree of precipitation reduction if the temperature increase is limited to 1.5°C instead of 2.0°C. If the Paris Agreement to limit global warming to 1.5°C will be accomplished, this will increase the probability for sustained viability of the *Stipa* steppes in the next 50–100 years (Guiot & Cramer, 2016; Mitchell et al., 2016; Pecl et al., 2017). It should be noted that this study used controlled experiments with seedlings of the four *Stipa* species under different temperature and precipitation treatments; thus, it is an important reference for understanding the growth of *Stipa* species under future climate changes. However, the growth of plants is comprehensively influenced by other factors, such as soil, topography, and human factors, so further studies with field observation experiments are also needed.

In conclusion, manipulation experiments were done under ambient and warming conditions with water variability, the response sensitivity of functional traits in the four *Stipa* species to water variability was relatively low in ambient temperature and increased in warming condition. R/S, SLA, and LA were the most response sensitive to water among all the functional traits and also made greater contribution to total biomass of the four grasses. The order of response sensitivity to water variability in the four species under ambient temperature treatment was ranked as follows: *S. bungeana* > *S. baicalensis* > *S. grandis* > *S. breviflora*, while *S. bungeana* had the greatest response sensitivity, and *S. grandis* had the lowest response sensitivity under warming treatments. The *Stipa* species were able to adapt better to mild decreased water when temperatures increased by 1.5°C rather than 2.0°C, indicating that future climate change will be more deleterious to *Stipa* growth (especially *S. bungeana*) if temperatures rise by 2.0°C rather than by 1.5°C. These findings contribute to our understanding of grassland species responses to global climate change and may be useful in providing experimental evidence for the Paris agreement.

## CONFLICT OF INTEREST

The authors declare no conflict of interest.

## AUTHOR CONTRIBUTIONS

GZ, QH, and XL designed the experiments. QH and XL conducted the experiments and collected the data; GZ, QH, and XL analyzed the data. XL wrote the manuscript; all authors contributed to manuscript editing and approved of the final version.

## ETHICAL APPROVAL

This study does not involve human participants or use of vertebrates.

## Data Availability

All the data used in this study will be accessible in Dryad data repository https://doi.org/10.5061/dryad.gm1h74f.

## References

[ece35452-bib-0001] Andresen, L. C. , Yuan, N. , Seibert, R. , Moser, G. , Kammann, C. I. , Luterbacher, J. , … Müller, C. (2017). Biomass responses in a temperate European grassland through 17 years of elevated CO_2_ . Global Change Biology, 24(9), 3875–3885. 10.1111/gcb.13705 28370878

[ece35452-bib-0002] Badger, M. R. , Björkman, O. , & Armond, P. A. (2006). Analysis of photosynthetic response and adaptation to temperature in higher plants: Temperature acclimation in the desert evergreen *Nerium Oleander* L. Plant Cell & Environment, 5, 85–99. 10.1111/1365-3040.ep11587620

[ece35452-bib-0003] Barkaoui, K. , Navas, M. , Roumet, C. , Cruz, P. , & Volaire, F. (2017). Does water shortage generate water stress? An ecohydrological approach across mediterranean plant communities. Functional Ecology, 31(6), 1325–1335. 10.1111/1365-2435.12824

[ece35452-bib-0004] Battisti, D. S. , & Naylor, R. L. (2009). Historical warnings of future food insecurity with unprecedented seasonal heat. Science, 323, 240–244. 10.1126/science.1164363 19131626

[ece35452-bib-0005] Bret‐Harte, M. S. , Shaver, G. R. , Zoerner, J. P. , Johnstone, J. F. , Wagner, J. L. , Chavez, A. S. , … Laundre, J. A. (2001). Developmental plasticity allows *Betula nana* to dominate tundra subjected to an altered environment. Ecology, 82, 18–32. 10.1890/0012-9658(2001)082[0018:Dpabnt]2.0.Co;2

[ece35452-bib-0006] Brodribb, T. J. , Bowman, D. M. J. S. , Nichols, S. , Delzon, S. , & Burlett, R. (2010). Xylem function and growth rate interact to determine recovery rates after exposure to extreme water deficit. New Phytologist, 188, 533–542. 10.1111/j.1469-8137.2010.03393.x 20673281

[ece35452-bib-0007] Brunner, I. , Herzog, C. , Dawes, M. A. , Arend, M. , & Sperisen, C. (2015). How tree roots respond to drought. Frontiers in Plant Science, 6, 547 10.3389/Fpls.2015.00547 26284083 PMC4518277

[ece35452-bib-0008] Castagneri, D. , Regev, L. , Boaretto, E. , & Carrer, M. (2017). Xylem anatomical traits reveal different strategies of two Mediterranean oaks to cope with drought and warming. Environmental and Experimental Botany, 133, 128–138. 10.1016/j.envexpbot.2016.10.009

[ece35452-bib-0009] Cavin, L. , Mountford, E. P. , Peterken, G. F. , & Jump, A. S. (2013). Extreme drought alters competitive dominance within and between tree species in a mixed forest stand. Functional Ecology, 27, 1424–1435. 10.1111/1365-2435.12126

[ece35452-bib-0010] Chapin, F. S. , Bloom, A. J. , Field, C. B. , & Waring, R. H. (1987). Plant responses to multiple environmental factors. BioScience, 37, 49–57. 10.2307/1310177

[ece35452-bib-0011] Cowling, R. M. , Potts, A. J. , Bradshaw, P. L. , Colville, J. , Arianoutsou, M. , Ferrier, S. , … Zutta, B. R. (2015). Variation in plant diversity in mediterranean‐climate ecosystems: The role of climatic and topographical stability. Journal of Biogeography, 42, 552–564. 10.1111/jbi.12429

[ece35452-bib-0012] Czajkowski, T. , Ahrends, B. , & Bolte, A. (2009). Critical limits of soil water availability (CL‐SWA) for forest trees–an approach based on plant water status. Landbauforsch Volkenrode, 59, 87–94.

[ece35452-bib-0013] De Boeck, H. J. , Lemmens, C. M. H. M. , Zavalloni, C. , Gielen, B. , Malchair, S. , Carnol, M. , … Nijs, I. (2008). Biomass production in experimental grasslands of different species richness during three years of climate warming. Biogeosciences, 4, 585–594. 10.5194/bg-5-585-2008

[ece35452-bib-0014] Donovan, L. A. , Maherali, H. , Caruso, C. M. , Huber, H. , & de Kroon, H. (2011). The evolution of the worldwide leaf economics spectrum. Trends in Ecology & Evolution, 26, 88–95. 10.1016/j.tree.2010.11.011 21196061

[ece35452-bib-0015] Fariaszewska, A. , Aper, J. , Van Huylenbroeck, J. , Baert, J. , De Riek, J. , Staniak, M. , & Pecio, L. (2017). Mild drought stress‐induced changes in yield, physiological processes and chemical composition in *Festuca*, *Lolium* and *Festulolium* . Journal of Agronomy and Crop Science, 203, 103–116. 10.1111/jac.12168

[ece35452-bib-0016] Guiot, J. , & Cramer, W. (2016). Climate change: The 2015 Paris Agreement thresholds and Mediterranean basin ecosystems. Science, 354, 465–468. 10.1126/science.aah5015 27789841

[ece35452-bib-0017] Hoover, D. L. , Knapp, A. K. , & Smith, M. D. (2016). Resistance and resilience of a grassland ecosystem to climate extremes. Ecology, 95, 2646–2656. 10.1890/13-2186.1

[ece35452-bib-0018] Hu, H. L. , Liu, Y. Z. , Li, Y. K. , Lu, D. X. , & Gao, M. (2014). Use of the N‐alkanes to estimate intake, apparent digestibility and diet composition in sheep grazing on *Stipa breviflora* desert steppe. Journal of Integrative Agriculture, 13, 1065–1072. 10.1016/S2095-3119(13)60502-X

[ece35452-bib-0019] Hu, X. W. , Zhou, Z. Q. , Li, T. S. , Wu, Y. P. , & Wang, Y. R. (2013). Environmental factors controlling seed germination and seedling recruitment of *Stipa bungeana* on the Loess Plateau of northwestern China. Ecological Research, 28, 801–809. 10.1007/s11284-013-1063-8

[ece35452-bib-0020] IPCC (2014). Climate change 2014: Impacts, adaptation, and vulnerability. Contribution of Working Groups I, II and III to the Fifth Assessment Report of the Intergovernmental Panel on Climate Change. Cambridge University Press, Cambridge, United Kingdom and New York, NY, USA.

[ece35452-bib-0021] Kang, L. , Han, X. G. , Zhang, Z. B. , & Sun, O. J. (2007). Grassland ecosystems in China: Review of current knowledge and research advancement. Philosophical Transactions of the Royal Society B‐Biological Sciences, 362, 997–1008. 10.1098/rstb.2007.2029 PMC2435566 17317645

[ece35452-bib-0022] Knapp, A. K. , Ciais, P. , & Smith, M. D. (2017). Reconciling inconsistencies in precipitation–productivity relationships: Implications for climate change. New Phytologist, 214, 41–47. 10.1111/nph.14381 28001290

[ece35452-bib-0023] Krishnamoorthy, K. , & Mathew, T. (2009). Statistical tolerance regions: Theory, applications, and computation. Hoboken, NJ: Wiley.

[ece35452-bib-0024] Lanari, V. , Silvestroni, O. , Palliotti, A. , Green, A. , & Sabbatini, P. (2015). Plant and leaf physiological responses to water stress in potted ‘Vignoles’ grapevine. HortScience, 50, 1492–1497. 10.21273/HORTSCI.50.10.1492

[ece35452-bib-0025] Lesk, C. , Rowhani, P. , & Ramankutty, N. (2016). Influence of extreme weather disasters on global crop production. Nature, 529, 84–87. 10.1038/nature16467 26738594

[ece35452-bib-0026] Li, F. , Zhao, W. Z. , & Liu, H. (2015). Productivity responses of desert vegetation to precipitation patterns across a rainfall gradient. Journal of Plant Research, 128, 283–294. 10.1007/s10265-014-0685-4 25613044

[ece35452-bib-0027] Li, G. , Han, H. , Du, Y. , Hui, D. , Xia, J. , Niu, S. , … Wan, S. (2017). Effects of warming and increased precipitation on net ecosystem productivity: A long‐term manipulative experiment in a semiarid grassland. Agricultural and Forest Meteorology, 232, 359–366. 10.1016/j.agrformet.2016.09.004

[ece35452-bib-0028] Li, Q. Y. , Xu, L. , Pan, X. B. , Zhang, L. Z. , Li, C. , Yang, N. , & Qi, J. G. (2016). Modeling phenological responses of Inner Mongolia grassland species to regional climate change. Environmental Research Letters, 11, 015002 10.1088/1748-9326/11/1/015002

[ece35452-bib-0029] Luers, A. L. (2005). The surface of vulnerability: An analytical framework for examining environmental change. Global Environmental Change‐Human and Policy Dimensions, 15, 214–223. 10.1016/j.gloenvcha.2005.04.003

[ece35452-bib-0030] Lv, X. , Zhou, G. , Wang, Y. , & Song, X. (2016). Sensitive indicators of zonal *Stipa* plants to changing temperature and precipitation in Inner Mongolia grassland. Frontiers in Plant Sciences, 7, 73 10.3389/fpls.2016.00073 PMC4744897 26904048

[ece35452-bib-0031] Ma, S. , Zhou, T. , Angélil, O. , & Shiogama, H. (2017). Increased chances of drought in southeastern periphery of the Tibetan plateau induced by anthropogenic warming. Journal of Climate, 30(16), 6543–6560. 10.1175/JCLI-D-16-0636.1

[ece35452-bib-0032] Manea, A. , Sloane, D. R. , & Leishman, M. R. (2016). Reductions in native grass biomass associated with drought facilitates the invasion of an exotic grass into a model grassland system. Oecologia, 181, 175–183. 10.1007/s00442-016-3553-1 26780256

[ece35452-bib-0033] Maréchaux, I. , Bartlett, M. K. , Sack, L. , Baraloto, C. , Engel, J. , Joetzjer, E. , & Chave, J. (2015). Drought tolerance as predicted by leaf water potential at turgor loss point varies strongly across species within an Amazonian forest. Functional Ecology, 29, 1268–1277. 10.1111/1365-2435.12452

[ece35452-bib-0034] Mitchell, D. , James, R. , Forster, P. M. , Betts, R. A. , Shiogama, H. , & Allen, M. (2016). Realizing the impacts of a 1.5 degrees C warmer world. Nature Climate Change, 6, 735–737. 10.1038/nclimate3055

[ece35452-bib-0035] Munson, S. M. , Muldavin, E. H. , Belnap, J. , Peters, D. P. C. , Anderson, J. P. , Reiser, M. H. , … Christiansen, T. A. (2013). Regional signatures of plant response to drought and elevated temperature across a desert ecosystem. Ecology, 94, 2030–2041. 10.1890/12-1586.1 24279274

[ece35452-bib-0036] Myneni, R. B. , Keeling, C. D. , Tucker, C. J. , Asrar, G. , & Nemani, R. R. (1997). Increased plant growth in the northern high latitudes from 1981 to 1991. Nature, 386, 698–702. 10.1038/386698a0

[ece35452-bib-0037] Nepstad, D. , Lefebvre, P. , Lopes da Silva, U. , Tomasella, J. , Schlesinger, P. , Solorzano, L. , … Guerreira Benito, J. (2004). Amazon drought and its implications for forest flammability and tree growth: A basin‐wide analysis. Global Change Biology, 10, 704–717. 10.1111/j.1529-8817.2003.00772.x

[ece35452-bib-0038] Palumbi, S. R. , Barshis, D. J. , Traylor‐Knowles, N. , & Bay, R. A. (2014). Mechanisms of reef coral resistance to future climate change. Science, 344, 895–898. 10.1126/science.1251336 24762535

[ece35452-bib-0039] Pecl, G. T. , Araújo, M. B. , Bell, J. D. , Blanchard, J. , Bonebrake, T. C. , Chen, I.‐C. , … Williams, S. E. (2017). Biodiversity redistribution under climate change: Impacts on ecosystems and human well‐being. Science, 355, 1389 10.1126/science.aai9214 28360268

[ece35452-bib-0040] Phillips, O. L. , Aragao, L. E. O. C. , Lewis, S. L. , Fisher, J. B. , Lloyd, J. , Lopez‐Gonzalez, G. , … Torres‐Lezama, A. (2009). Drought sensitivity of the Amazon Rainforest. Science, 323, 1344–1347. 10.1126/science.1164033 19265020

[ece35452-bib-0041] Qi, Y. C. , Dong, Y. S. , Liu, L. X. , Liu, X. R. , Peng, Q. , Xiao, S. S. , & He, Y. T. (2010). Spatial‐temporal variation in soil respiration and its controlling factors in three steppes of *Stipa* L. in Inner Mongolia, China. Science China‐Earth Sciences, 53, 683–693. 10.1007/s11430-010-0039-6

[ece35452-bib-0042] Reichstein, M. , Bahn, M. , Ciais, P. , Frank, D. , Mahecha, M. D. , Seneviratne, S. I. , … Wattenbach, M. (2013). Climate extremes and the carbon cycle. Nature, 500, 287–295. 10.1038/nature12350 23955228

[ece35452-bib-0043] Salgado‐Negret, B. , Canessa, R. , Valladares, F. , Armesto, J. J. , & Perez, F. (2015). Functional traits variation explains the distribution of *Aextoxicon punctatum* (*Aextoxicaceae*) in pronounced moisture gradients within fog‐dependent forest fragments. Frontiers in Plant Science, 6, 511 10.3389/fpls.2015.00511 26257746 PMC4511825

[ece35452-bib-0044] Sankaran, M. , Hanan, N. P. , Scholes, R. J. , Ratnam, J. , Augustine, D. J. , Cade, B. S. , … Zambatis, N. (2005). Determinants of woody cover in African savannas. Nature, 438, 846–849. 10.1038/nature04070 16341012

[ece35452-bib-0045] Shafran‐Nathan, R. , Svoray, T. , & Perevolotsky, A. (2013). The resilience of annual vegetation primary production subjected to different climate change scenarios. Climatic Change, 118, 227–243. 10.1007/s10584-012-0614-2

[ece35452-bib-0046] Shi, Y. , Zhou, G. , Jiang, Y. , Wang, H. , & Xu, Z. (2016). Does precipitation mediate the effects of elevated Co_2_, on plant growth in the grass species *Stipa grandis* . Environmental & Experimental Botany, 131, 146–154. 10.1016/j.envexpbot.2016.07.011

[ece35452-bib-0047] Song, X. , Wang, Y. , & Lv, X. (2016). Responses of plant biomass, photosynthesis and lipid peroxidation to warming and precipitation change in two dominant species (*Stipa grandis* and *Leymus chinensis*) from North China Grasslands. Ecology and Evolution, 6(6), 1871–1882. 10.1002/ece3.1982 26933491 PMC4760990

[ece35452-bib-0048] Sui, X. H. , Zhou, G. S. , & Zhuang, Q. L. (2013). Sensitivity of carbon budget to historical climate variability and atmospheric CO_2_ concentration in temperate grassland ecosystems in China. Climatic Change, 117, 259–272. 10.1007/s10584-012-0533-2

[ece35452-bib-0049] Sun, H. , Wang, Y. , Chen, J. , Zhai, J. , Jing, C. , Zeng, X. , … Su, B. (2017). Exposure of population to droughts in the haihe river basin under global warming of 1.5 and 2.0°C scenarios. Quaternary International, 453, 74–84. 10.1016/j.quaint.2017.05.005

[ece35452-bib-0050] Tan, L. , Cai, Y. , An, Z. , Cheng, H. , Shen, C. C. , Gao, Y. , & Edwards, R. L. (2017). Decreasing monsoon precipitation in southwest china during the last 240 years associated with the warming of Tropical Ocean. Climate Dynamics, 48, 1–10. 10.1007/s00382-016-3171-y

[ece35452-bib-0051] Thompson, R. B. , Gallardo, M. , Valdez, L. C. , & Fernández, M. (2007). Using plant water status to define threshold values for irrigation management of vegetable crops using soil moisture sensors. Agricultural Water Management, 88, 147–158. 10.1016/j.agwat.2006.10.007

[ece35452-bib-0052] Waddington, J. M. , Morris, P. J. , Kettridge, N. , Granath, G. , Thompson, D. K. , & Moore, P. A. (2015). Hydrological feedbacks in northern peatlands. Ecohydrology, 8, 113–127. 10.1002/eco.1493

[ece35452-bib-0053] Wertin, T. M. , Reed, S. C. , & Belnap, J. (2015). C3 and c4 plant responses to increased temperatures and altered monsoonal precipitation in a cool desert on the Colorado Plateau, USA. Oecologia, 177, 997–1013. 10.1007/s00442-015-3235-4 25676102

[ece35452-bib-0054] Wilcox, K. R. , von Fischer, J. C. , Muscha, J. M. , Petersen, M. K. , & Knapp, A. K. (2015). Contrasting above‐ and belowground sensitivity of three Great Plains grasslands to altered rainfall regimes. Global Change Biology, 21, 335–344. 10.1111/gcb.12673 25044242

[ece35452-bib-0055] Wright, I. J. , Reich, P. B. , Westoby, M. , Ackerly, D. D. , Baruch, Z. , Bongers, F. , … Villar, R. (2004). The worldwide leaf economics spectrum. Nature, 428, 821–827. 10.1038/nature02403 15103368

[ece35452-bib-0056] Wu, Z. , Dijkstra, P. , Koch, G. W. , Peñuelas, J. , & Hungate, B. A. (2011). Responses of terrestrial ecosystems to temperature and precipitation change: A meta‐analysis of experimental manipulation. Global Change Biology, 17, 927–942. 10.1111/j.1365-2486.2010.02302.x

[ece35452-bib-0057] Xu, X. , Zhao, S. , Yin, Y. , Xie, T. , Yu, Z. , Fang, X. , & Ding, W. (2009). Statistical interpretation of data determination of statistical tolerance intervals (GB/T 3359–2009/ISO 16269–6:2005). Shanxi, China: China Standards Press.

[ece35452-bib-0058] Xu, Z. Z. , Shimizu, H. , Ito, S. , Yagasaki, Y. , Zou, C. J. , Zhou, G. S. , & Zheng, Y. R. (2014). Effects of elevated CO_2_, warming and precipitation change on plant growth, photosynthesis and peroxidation in dominant species from North China grassland. Planta, 239, 421–435. 10.1007/s00425-013-1987-9 24463932

[ece35452-bib-0059] Xu, Z. Z. , & Zhou, G. S. (2011). Responses of photosynthetic capacity to soil moisture gradient in perennial rhizome grass and perennial bunchgrass. BMC Plant Biology, 11, 21 10.1186/1471-2229-11-21 21266062 PMC3037845

[ece35452-bib-0060] Yang, Y. , Wang, G. , Yang, L. , & Guo, J. (2013). Effects of drought and warming on biomass, nutrient allocation, and oxidative stress in *Abies fabri* in eastern Tibetan Plateau. Journal of Plant Growth Regulation, 32, 298–306. 10.1007/s00344-012-9298-0

[ece35452-bib-0061] Young, D. S. (2014). Computing tolerance intervals and regions using R. Handbook of Statistics, 32, 309–338. 10.1016/B978-0-444-63431-3.00008-5

[ece35452-bib-0062] Zhou, G. , He, Q. , & Yin, X. (2015). Adaptability and vulnerability of Chinese vegetation/terrestrial ecosystem to climate change. Beijing, China: Meteorological Press. (In Chinese).

